# Ultra-responsive soft matter from strain-stiffening hydrogels

**DOI:** 10.1038/ncomms6808

**Published:** 2014-12-16

**Authors:** Maarten Jaspers, Matthew Dennison, Mathijs F. J. Mabesoone, Frederick C. MacKintosh, Alan E. Rowan, Paul H. J. Kouwer

**Affiliations:** 1 Department of Molecular Materials, Radboud University Nijmegen, Institute for Molecules and Materials, Heyendaalseweg 135, 6525 Nijmegen, The Netherlands; 2 Department of Physics and Astronomy VU University, De Boelelaan, 1081 Amsterdam, The Netherlands

## Abstract

The stiffness of hydrogels is crucial for their application. Nature’s hydrogels become stiffer as they are strained. This stiffness is not constant but increases when the gel is strained. This stiffening is used, for instance, by cells that actively strain their environment to modulate their function. When optimized, such strain-stiffening materials become extremely sensitive and very responsive to stress. Strain stiffening, however, is unexplored in synthetic gels since the structural design parameters are unknown. Here we uncover how readily tuneable parameters such as concentration, temperature and polymer length impact the stiffening behaviour. Our work also reveals the marginal point, a well-described but never observed, critical point in the gelation process. Around this point, we observe a transition from a low-viscous liquid to an elastic gel upon applying minute stresses. Our experimental work in combination with network theory yields universal design principles for future strain-stiffening materials.

## Abstract (web summary)

Few synthetic hydrogels are known to display strain-stiffening behaviour. Here, Jaspers *et al.* show how concentration, polymer length and temperature can be used to modify the mechanical properties of synthetic gels to access mechanically highly sensitive and responsive materials.

## Graphical abstract

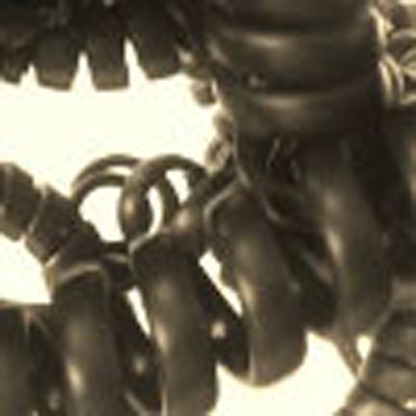

Mechano-responsive gels are very common in nature; for instance, gels based on actin, collagen, fibrin, intermediate filaments and many more proteins and carbohydrates display mechanical properties that are not only dependent on their biochemical environment, but also show an immediate response to deformation^1^,^2^. Many of these gels strain-stiffen (they become stiffer as the stress or strain in the material increases), which, for instance, aids in the protection of tissues from rupture and in long-distance cell–cell communication^1^,^3^. Typically, these biopolymers show a universal structural design element: they are relatively stiff and assemble into bundles or fibrils of defined dimensions, which results in both a high sensitivity and a high responsiveness towards stress.

In contrast to these biogels, synthetic hydrogels, including many artificial extracellular matrices are generally not (or at most very weakly) responsive to stress, that is, their stiffness is constant over the entire relevant stress range^4^,^5^. If stiffening behaviour is observed at all, it is found at high stresses; for (soft) hydrogels, this is commonly far beyond the rupture stress of the material. It is surprising that, despite the general consensus on the crucial role of mechanics in biomedical processes^5^,^6^,^7^,^8^,^9^,^10^, the stiffening aspect, and in particular the difference in behaviour of biological and synthetic gels has remained largely overlooked^11^,^12^,^13^. As a result, the relation between common network parameters and conditions with mechanical responsiveness are only available from studies on biopolymer gels^1^,^2^,^14^ (with highly limited parameter space) or from theory and simulations^15^.

Recently, we reported a fully synthetic gel that uniquely stiffens in the same stress and strain regime as biogels^16^. In this paper we exploit the broad scope for manipulation that synthetic materials offer, and we describe the effect of common, easily controllable variables (concentration, polymer length and temperature) on the mechanical properties of semi-flexible polymer hydrogels. We show for instance that at sufficient stress, a universal mechanical response is observed, which means that sometimes, counterintuitively, stiffer materials can be obtained at lower polymer concentrations. Moreover, the thermoresponsive nature of our gel allows us to precisely control the crosslink density, a tool to enter the marginal regime that is the regime where the fluid polymer solution turns into an elastic gel. Our work in this experimentally unexplored regime agrees quantitatively with theory and simulation results of semi-flexible networks^17^. This excellent agreement emphasizes that our results are not specific for our polymer gels only, but will be generally applicable for the design of other mechano-responsive soft matter.

## Results

### Materials

**Figure 1:**
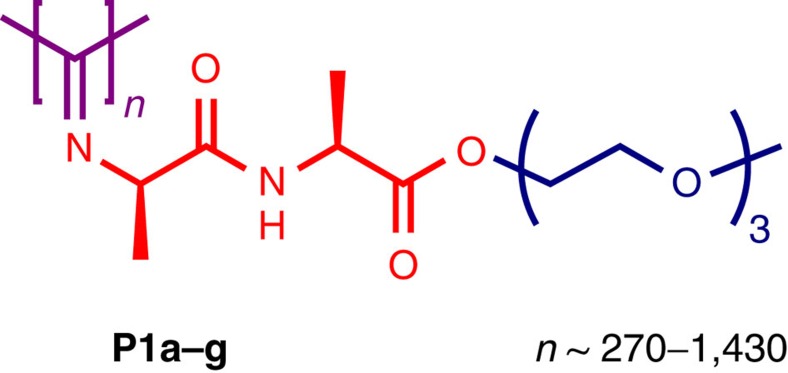
Ethylene glycol substituted polyisocyanopeptides. Molecular structure of the tri(ethylene glycol)-substituted polyisocyanopeptides. In purple the polymer backbone of 270–1,430 repeat units. Every carbon atom holds a substituent, composed of a dipeptide (Ala–Ala, red) and a short methyl-terminated tri(ethylene glycol) tail (blue).

The biomimetic strain-stiffening hydrogels are based on ethyleneglycol-functionalized polyisocyanopeptides **P1a–g** (PICs, Fig. 1). The polymers were synthesized and characterized using a slightly modified literature procedure (see Methods section)^16^,^18^. Variation of the monomer/catalyst ratio in the reaction mixture gave control over the polymer length (Table 1), which was characterized by viscometry and atomic force microscopy (AFM) studies (Supplementary Fig. 1) since standard polymer length characterization techniques based on separation by gel permeation chromatography or field-flow fractionation gave unreliable results (poor separation), possibly due to the high backbone stiffness. We used viscometry data (available over the entire molecular weight range) in combination with the Mark−Houwink equation (Methods section) to determine molecular weights and with AFM data to determine distributions. As such, we prepared seven polymers with molecular weights ranging from *M*_v_=80–450 kg mol^−1^, equivalent to average contour lengths *L* of 30–180 nm (ref. 19). To prepare a hydrogel, a small amount of polymer (typically 1 mg) is dissolved in cold water (1 ml) by stirring for 24 h in a cold room at 4 °C and subsequently heating the solution beyond the gelation temperature, which is polymer length and (weakly) concentration dependent. The thermoreversible gelation process is the result of hydrophobic effects of the oligo(ethyleneglycol) tails^20^. At the lower critical solution temperature, the polymer becomes hydrophobic and forms the entangled bundles of polymer chains of which the hydrogel is composed.

### Mechanical analysis

**Figure 2:**
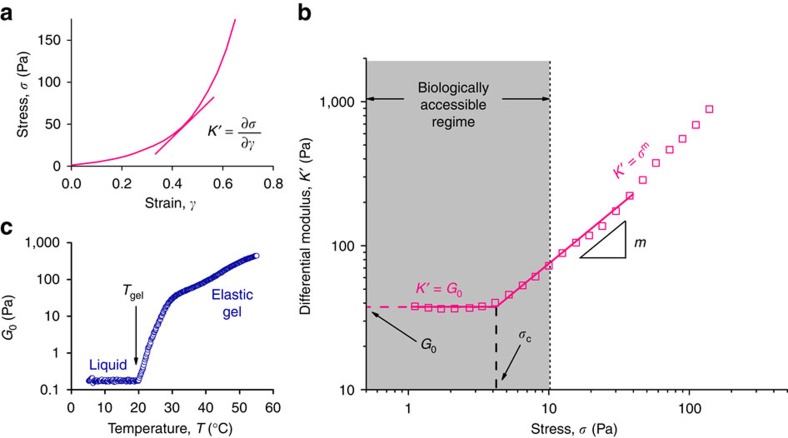
Strain stiffening of a polyisocyanopeptide hydrogel. (**a**) Stress-strain curve of polymer **P1f** (1 mg ml^−1^, *T*=37 °C) in a stress ramp. (**b**) The stiffness represented as the differential modulus *K*′≡δ*σ*/δ*γ* as a function of stress *σ* for the same polymer. At low stress, *K*′=*G*_0_ the plateau modulus, but beyond a critical stress *σ*_c_, *K*′ increases, following *K*′∝*σ*^*m*^ where the exponent *m* is the stiffening index. (**c**) The thermally induced gelation process is unmistakably observed by plotting *G*_0_ versus temperature *T*. We define the gel point as the onset of modulus increase, here at 19 °C.

In strain-stiffening materials, the plateau shear modulus *G*_0_, defined as the ratio between the stress *σ* and the strain *γ*, is not a constant but increases with increasing stress (or strain) after a critical stress *σ*_c_ is reached (Fig. 2a). To more accurately describe the mechanical behaviour, we use the differential modulus *K*′=δ*σ*/δ*γ* and define a low stress linear regime where *K*′=*G*_0_ and a higher stress stiffening regime where the modulus depends on *σ* (Fig. 2b). This introduces two additional important parameters: the critical stress *σ*_c_ and stiffening index *m* (Fig. 2b). The former is the stress onset for nonlinearity and determines the sensitivity of the material; a low *σ*_c_ yields a high mechanical sensitivity. The latter index *m* represents the (intensity of the) stress response. Indeed, biological gels are commonly characterized by these three parameters. For instance, a typical actin gel has a high plateau modulus, a low critical stress (very sensitive to small stresses) and a high stiffening index (large response)^2^. Contrarily, a fibrin gel at similar concentration has a much lower *G*_0_, a higher *σ*_c_ and a stiffening index that is a function of the applied stress resulting from the hierarchical organization of the protein assembly^21^.

The experimental data of the **P1f** gel (Fig. 2a,b) shows that for the PIC hydrogels, *σ*_c_ lies well into the biologically accessible range^13^ (the forces that cells can apply to their matrix) and that the response to stress and other external factors such as temperature (Fig. 2c) is large. In the following paragraphs, we demonstrate how the mechanical parameters stiffness *G*_0_, sensitivity *σ*_c_ and responsiveness *m* can be controlled by the variables temperature *T*, polymer length *L* and concentration *c*. Can one, for instance, independently tune *G*_0_ and *σ*_c_ to give stiffer, but nonetheless more sensitive materials? How do *T*, *L* and *c* impact the strength of the response? Or more generally, what are the important design parameters to create highly mechanically responsive materials and how should one access these highly responsive regimes?

We performed macroscopic rheology to determine the mechanical properties of the hydrogels in the linear and the nonlinear regime. The nonlinear regime was studied using a pre-stress protocol, where the sample at the desired temperature was subjected to a constant stress with a small oscillatory stress superposed. We compare our experimental results to a model designed to describe the linear and nonlinear mechanical properties of semi-flexible networks^2^,^22^. The model we use assumes no network relaxation over the timespan of the experiment. Chemically crosslinked networks commonly hold to this assumption, but for physically crosslinked gels this is not always the case. We experimentally verified by low-frequency rheological measurements that the PIC gel does not relax on a time scale 10 times the measurement time, even in very soft regimes, close to the gel point. This model describes *G*_0_ and *σ*_c_ quantitatively as a function of the polymer (or filament) concentration *c* and persistence length *l*_p_, the temperature *T* and the distance between crosslinks in the network *l*_c_:


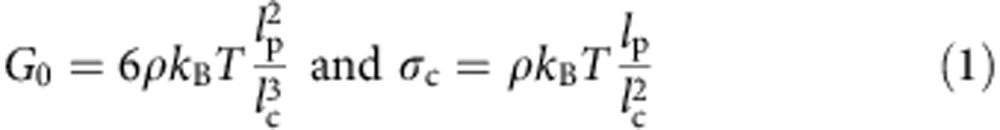


where the polymer density in length per volume *ρ*=*χc*/*N* and *χ* includes molecular constants^16^, *N* is the number of polymer chains per bundle and *k*_B_ is Boltzmann’s constant. PIC networks show a finite bundling behaviour in the gel (independent of concentration) and a thermal stiffening of the polymer chains. At our default gel concentrations (*c*=0.1–5 mg ml^−1^) where *l*_c_ approximates the mesh size, equation (1) can be approximated as:


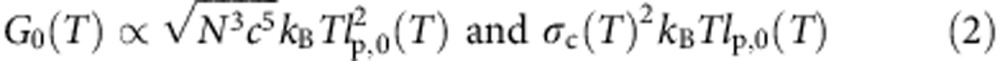


where *l*_p,0_ is the persistence length of a single polymer chain rather than the bundle^16^.

### Concentration dependence

**Figure 3:**
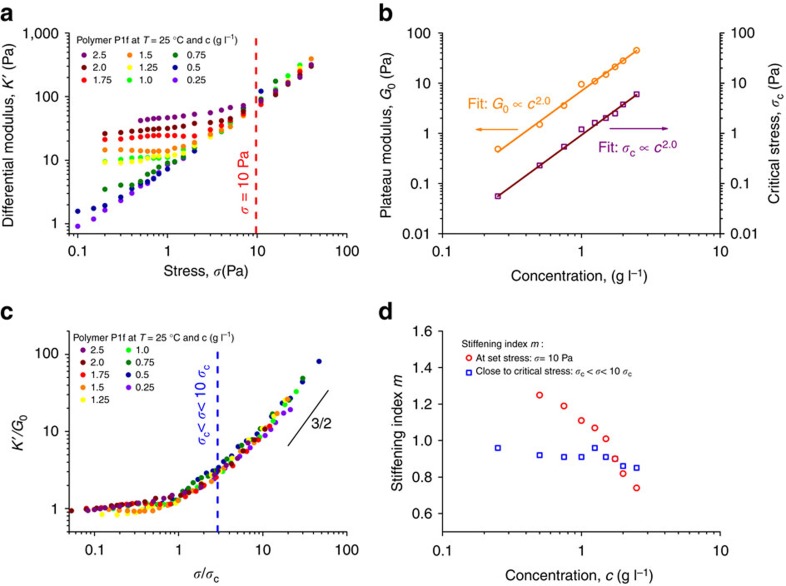
Mechanical properties of PIC hydrogels (P1f, *T*=25 °C) as a function of polymer concentration. (**a**) Differential modulus against stress at different polymer concentrations. Note that the *y*-axis spans 3 orders of magnitude in stiffness with a concentration range of only 1 order of magnitude. The dashed red line at *σ*=10 Pa indicates where *m* was determined. (**b**) Plateau modulus *G*_0_ (orange) and critical stress *σ*_c_ (dark red) against concentration (**c**). The solid lines are power law fits and show that both scale with *c*^2.0^. (**c**) Scaling *K*′ with *G*_0_ and *σ* with *σ*_c_ causes a collapse of the data to a single master curve that shows *K*′∝*σ*^3/2^ only at high *σ*. The blue dashed line shows the point close to *σ*_c_ where *m* was determined. (**d**) Stiffening index *m* as a function of concentration. When determined close to *σ*_c_ (blue squares), *m* is similar for all concentrations; when determined at a given stress (red circles, *σ*=10 Pa), *m* is higher for lower concentration gels (since at lower *c*, *σ*_c_ is much lower and thus *σ*–*σ*_c_ will be higher). Note that the concentration axes (**b**,**d**) have logarithmic scales.

The easiest method to change the mechanical properties of any hydrogel is to vary the concentration *c* of the constituent polymer. A concentration study of **1f** (*L*=158 nm) reveals that, indeed, the plateau modulus *G*_0_ and critical stress *σ*_c_ are strongly dependent on *c* (Fig. 3). Plotting the differential modulus *K′* against stress as a function of concentration (Fig. 3a) clearly shows for all samples a linear regime at low *σ* and a nonlinear regime at higher *σ*. In addition, the absolute values of *G*_0_ and *σ*_c_ increase with *c*; scaling analysis shows a square dependence with *c* for both (Fig. 3b), which corresponds to theoretical and literature values^2^,^22^,^23^. The curves collapse to a master curve when scaled against *G*_0_ and *σ*_c_ (Fig. 3c). The plot suggests that a stiffening index *m*=3/2 is reached only at high stresses, as was observed previously at elevated temperatures^16^. The 3/2 exponent is the upper limit for *m* and is associated to enthalpically stretching a polymer chain along its length. Data recorded at 25 °C, only a few degrees away from *T*_gel_, however, showed lower stiffening indices. For all concentrations, *m*≈1 close to *σ*_c_ (blue line Fig. 3c and blue squares Fig. 3d), while *m* increases at higher stresses. Hence, at a specific stress, for instance *σ*=10 Pa (red line Fig. 3a), *m* decreases for samples with increasing *σ*_c_ (closer to 10 Pa) and thus with increasing concentration (red circles Fig. 3d). In other words, not only does the gel become less mechanically sensitive at higher concentration, also its stress response becomes weaker.

This seemingly straightforward series of experiments yields two important insights. First, at high stress all gels have similar stiffnesses, nearly independent of the concentration. This is particularly important for biomedical applications of hydrogels, where both morphology and stiffness are key for their application. When cells stiffen their own matrix^13^, an increase in concentration may not contribute to an increase in the mechanical (micro)environment. Second, the concentration as a variable equally affects the plateau modulus and the critical stress, that is, hydrogels with higher elastic moduli inevitably become less sensitive to stress. This renders concentration a relatively ineffective tool to flexibly design the mechanical properties of a gel.

### Chain length dependence

**Figure 4:**
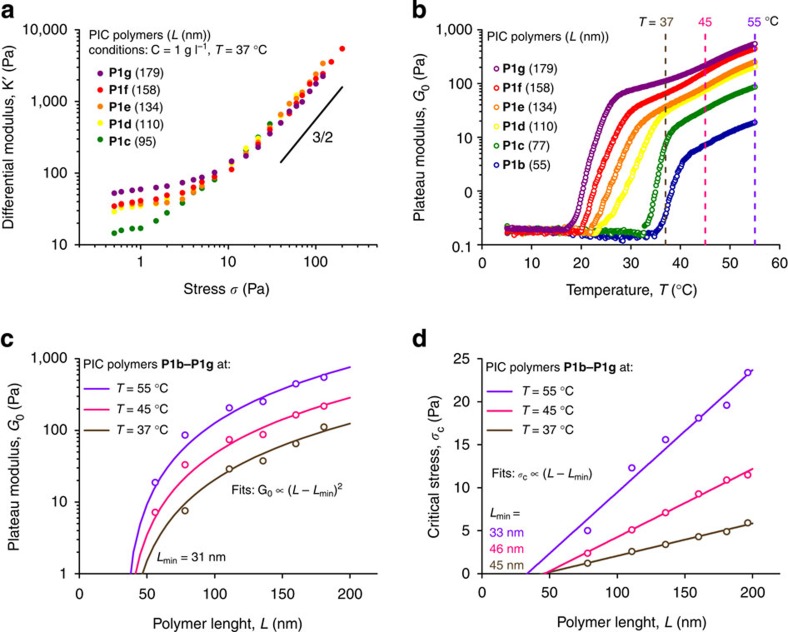
Molecular weight dependence of the mechanical properties of the P1b–P1g hydrogels (at *c*=1 mg ml^−1^). (**a**) Differential modulus against stress for different molecular weight or length *L* polymers at *T*=37 °C. The solid line shows the high stress limit *m*=3/2. (**b**) Plateau modulus *G*_0_ as a function of temperature for gels of different length polymers. (**c**) Data extracted from **b**: *G*_0_ as a function of *L* at *T*=37, 45 and 55 °C. The solid line shows quadratic fits to (*L*–*L*_min_), where fitting parameter *L*_min_ represents a minimum polymer length (see main text). (**d**) The critical stress *σ*_c_ as a function of *L* at the same temperature. Here the solid lines are linear fits to (*L*–*L*_min_).

Another readily accessible variable available to polymer chemists is the molecular weight or the length of the polymers. Polymers **1a–1g**, obtained by polymerizations with different monomer/catalyst ratios have contour lengths *L*=20–180 nm (Table 1) with similar chain length distributions (Supplementary Fig. 1). We experimentally investigated the effect of the polymer chain length on the linear and nonlinear mechanical properties of the hydrogels, keeping the concentration of the polymers constant at *c*=1 mg ml^−1^ (Fig. 4).

For covalently crosslinked gels of either flexible or semi-flexible polymers, the critical length scale that determines the stiffness is not the chain length, but the length between crosslinks *l*_c_, which is predominantly controlled by the polymer concentration and the crosslink density. Recent computational simulations on semi-flexible polymer gels, however, indicated that the gel stiffness will also depend on chain contour length *L* for relatively short and flexible polymers, while for longer or stiffer polymers no dependency was observed^24^. Experimentally, we observe that the PIC gels require a minimum length to form gels of sufficient strength to investigate linearly or nonlinearly. Gels of polymer **P1a** were too weak to measure reliably, and gels of **P1b** ruptured at increased stress. Polymers **P1c**–**P1g**, however, show a clear linear and nonlinear regime (Fig. 4a), where both the (linear) stiffness *G*_0_ and critical stress *σ*_c_ increase with increasing *L*. Again, at high stress, all polymer gels behave similarly. With the polymer length, also the gelation temperature changes (Fig. 4b), where the shorter polymers have an increasing gel temperature. The increase in *T*_gel_, is tentatively attributed to a delayed bundling process. At three different temperatures (dashed lines in Fig. 4b), *G*_0_ (Fig. 4c) and *σ*_c_ (Fig. 4d) were recorded for the different length polymers. The observed dependencies: *G*_0_∝*L*^2^ and *σ*_c_∝*L* are in perfect agreement with theoretical predictions for relatively short semi-flexible polymers^24^. Only good fits, however, are obtained when a minimum cut-off length *L*_min_≈35 nm is considered, indicating a lower length limit for efficient bundle formation. We emphasize that *L*_min_ is unrelated to the mesh size of the gel at this concentration, which for these materials is 3–4 times larger. The inability of **P1a** (with average polymer length *L*=34 nm) to form hydrogels supports the model of a minimum required polymer length. The different scaling response to *G*_0_ and *σ*_c_ makes *L* (compared to *c*) a far more effective parameter to tune the mechanical properties of a gel.

### Temperature dependence

**Figure 5:**
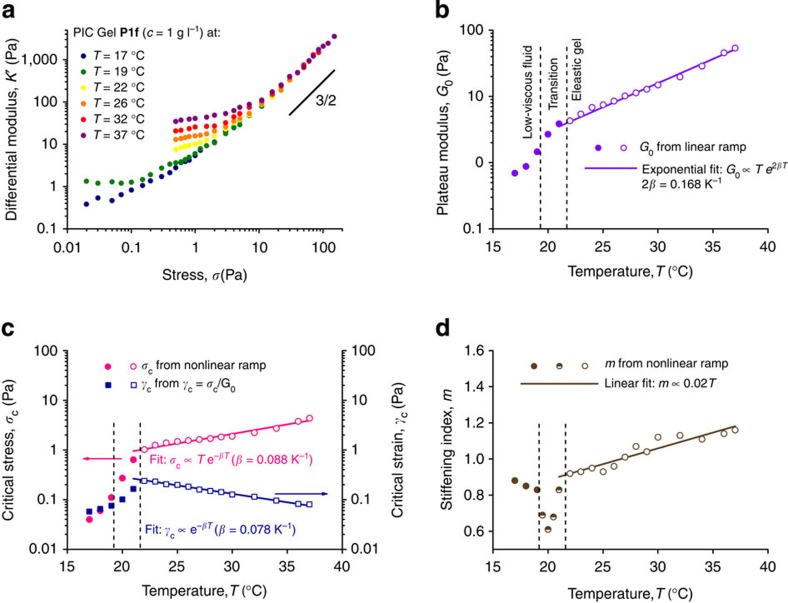
Temperature dependence of the mechanical properties of the P1f hydrogel (at 1 mg ml^–1^). (**a**) Differential modulus against stress at different temperatures. The solid line shows the high stress limit *m*=3/2. (**b**) Plateau modulus *G*_0_, as a function of temperature *T* (linear scale). In the gel at *T*≥22 °C, *G*_*0*_ increases exponentially with *T* over the investigated temperature window; the solid line is a fit to *Te*^2*βT*^ with *β*≈0.08 K^−1^. At *T*≤22 °C, *G*_0_ increases fast with *T*, corresponding to the transition from liquid to gel. (**c**) critical stress *σ*_c_ (pink circles) and critical strain *γ*_c_ (blue squares) as a function of *T*; the solid lines are fits to *Te*^*βT*^ and *e*^−*βT*^ with *β*≈0.08 K^−1^. Both *σ*_c_ and *γ*_c_ show a sharp decrease at *T*≤22 °C. (**d**) The stiffening index *m* as a function of *T* clearly shows three regimes. In the high temperature regime, *m* weakly scales linearly with *T*, and around the marginal regime (see text) *m* sharply decreases while it restores to 0.9 on further decreasing *T*. The values for *m* were recorded at stresses close to the critical stress (at *σ*=3*σ*_c_). At higher stress, in particular at high temperature, *m* will approach 1.5 ref. 16, but such stresses are impossible to apply close to *T*_gel_.

The largest change in properties in PIC-based gels is thermally induced. The PIC polymers form low-viscosity solutions at low temperatures and elastic gels when heated beyond their gelation temperature. Hydrophobic interactions of the oligoglycol substituents are responsible for this lower critical solution temperature behaviour, which has also been observed in other polymers^20^. All mechanical properties change drastically in the (reversible) transition from a liquid to a hydrogel and we will discuss three regimes separately: in the gel (high *T*), in the transition and just below the transition (low *T*). The overall mechanical properties show that on increasing the temperature both the stiffness and the critical stress increase (Fig. 5a). At high stress, however, the data at different temperatures all collapse to a single curve, similar as was the case in the concentration and length studies.

The stiffness *G*_0_ in the elastic gel regime shows a dual thermal response (Fig. 5b). Network theory predicts a linear dependence on *T*, associated with thermal fluctuations of the polymer chains. When the thermal fluctuations increase, it becomes more difficult to stretch the polymer chains and hence, the stiffness increases^22^. A second, exponential contribution originates from the stiffening of the polymer chains (increase in *l*_p,0_) themselves with *T*, as was established earlier for PIC gels^16^. Both factors together contribute with:


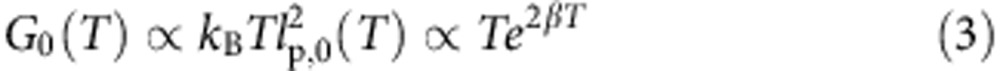


For **P1f**, we find *β*≈0.084 K^−1^, which means that the gel stiffness doubles every ~4 °C. In and below the gelation transition, *G*_0_ decreases rapidly until at low temperatures it becomes difficult to measure *G*_0_ accurately due to the limitations of our experimental set-up.

The critical stress similarly depends on *T* and *l*_p,0_(*T*) and is expected to scale as *k*_B_*Te*^*βT*^, as was also observed experimentally (Fig. 5c, pink data) with a similar exponent *β*=0.088 K^−1^. The temperature increases the stiffness more than the critical stress, which results in a decrease in the critical strain *γ*_c_ with *T*:


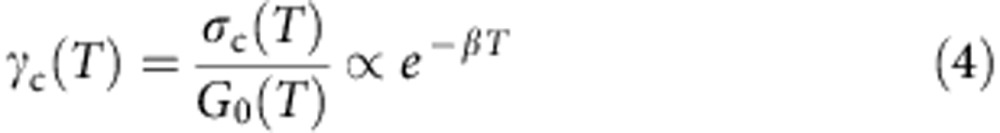


Indeed, we see experimentally (Fig. 5c, blue data) that at elevated temperatures only very small deformations (*γ*_c*,T*=37_ _°C_=8%) are required to enter the nonlinear, responsive regime, while at lower *T* these deformations must be much higher (*γ*_c*,T*=22_ _°C_=25%). The absolute value of the critical strain in a semi-flexible gel can further be tuned by the polymer length *L*, where for increased *L* a decrease in *γ*_c_ is expected based on equation (4). Again, changing the polymer concentration will be less effective as both *σ*_c_ and *G*_0_ scale similarly with *c* (Fig. 3b). The exponential decrease in *γ*_c_ given by *β*, however, is a molecular property that cannot be altered by changing *L* or *c*. In contrast to *G*_0_, *σ*_c_ and *γ*_c_, *m* is only weakly temperature dependent in the gel regime (Fig. 5d).

We also measured the mechanical properties of the aqueous materials during the gelation transition (*T*=19–22 °C) and even before gelation, when the material is a low-viscous dilute polymer solution (*T*=17–19 °C). At even lower temperatures, instrument inertia dominates our rheological data, which renders our results unreliable. In the entropically driven phase transition, the connectivity (that is, the number of crosslinks) of the network will rapidly change as a function of *T*. This causes *G*_0_ to increase by a factor of 10 over only 5 °C (Fig. 4b) and *σ*_c_ even more (Fig. 4c). As a result, *γ*_c_ shows a maximum precisely at the temperature where the network is fully established. This is an important factor in designing soft, highly strainable networks, as the stiffness of the materials rapidly increases when strained beyond the critical strain.

The stiffening index *m*, the strength of the mechanical response to stress shows two additional regimes. When cooling from 22 °C, it rapidly decreases to *m*≈0.6. By decreasing the connectivity in a network, simulations have found anomalous behaviour at the critical point, which is the connectivity at which the network becomes mechanically floppy. This point, called the marginal point, is predicted to show a decreased stiffening index (*m*=0.5). So far, marginal materials have been described only in theory and by simulations^17^ and no experimental (bio)polymer network has validated these predictions yet. The PIC gels are the first materials that display some of the predicted characteristics^25^. An additional prediction is a vanishing *σ*_c_ (also experimentally observed in the PIC hydrogel, Fig. 5c), which makes the material in and around the marginal point extremely stress sensitive and allows the design of materials that in a small stress (or strain) regime can span 2 or 3 orders of magnitude in stiffness.

Upon further lowering *T*, *m* increases again to ~0.9. In this ‘pre-marginal’ regime, no continuous network is yet formed, but stress applied to the semi-flexible polymer chains induces network formation. Simulations show that *m* should reach unity eventually, but we were unable to experimentally probe deeper in the pre-marginal regime. The material at this temperature range has extremely interesting properties. At low stress or deformation, it behaves as a fluid; it can be poured from a vial. Rheologically, we see that the viscous properties dominate the elastic properties (Supplementary Fig. 2). However, as the nonlinear strain-stiffening behaviour is still present in the material, the elasticity increases steeply with stress. Since, in addition, the critical stress is very small (*σ*_c,*T*=17_ _°C_<0.05 Pa), the threshold for stiffening is virtually absent and the solution transforms into a fully elastic solid at very low stress. Finally, the relatively high stiffening index in this regime causes the stiffness to increase rapidly on further increasing the stress. Even at relatively low stresses, for example, at 1 Pa, *K*′ already increased by an order of magnitude. This makes marginal, but even more so pre-marginal, ‘gels’ or solutions extremely sensitive and also very responsive to stress. Such materials could also be interesting for tissue engineering applications when one considers that cells can easily apply forces of such magnitude to their environment, and thus can manipulate the local mechanical properties of their matrix.

## Discussion

**Figure 6:**
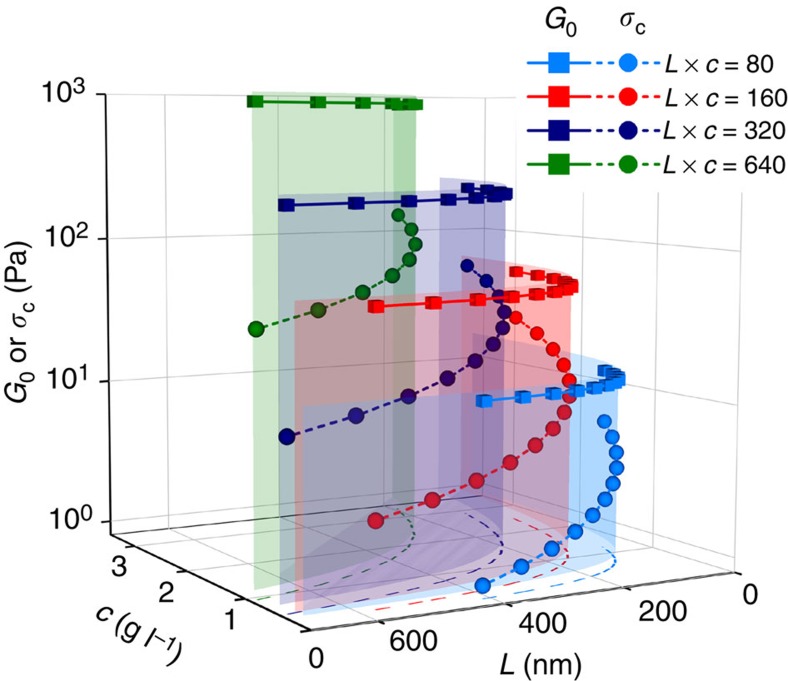
Interpolated mechanical properties of gels with constant modulus and varying sensitivity to stress. When in a series of gels the product *L* × *c* and *G*_0_ remain constant, but *σ*_c_ changes. Here we selected four series of constant *G*_0_ in the range 10–1,000 Pa (squares) and *σ*_c_ varying in each series (circles). The projection of the curves on the *x*–*y* plane (dashed lines) shows the corresponding length and concentrations.

The controlled manipulation of the linear and nonlinear mechanical properties of a hydrogel is a complex task. Changing a variable such as concentration affects all three mechanical parameters *G*_0_, *σ*_c_ and *m*. In addition, important biological parameters, such as pore size also change. This makes it difficult to make appropriate comparisons within a series, let alone between series. A thorough study of these different dependencies, however, allows one to profit from this complex behaviour. As an example, the stronger increase of *G*_0_ (quadratic) compared with *σ*_c_ (linear) with *L* presents an opportunity to develop specific materials properties, such as series of materials with constant stiffness, but with different sensitivity (and response) to deformation. When keeping *L* × *c* constant, *G*_0_ remains constant, but *σ*_c_ varies with their ratio (Fig. 6). Changing the value of *L* × *c* results in different (constant) linear moduli, but in the same overall behaviour. Analogously, one can prepare series of materials with the same critical stress (keeping 
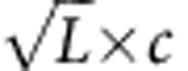
 constant) but by varying linear modulus. This example of a combined length–concentration approach allows for full control on stiffness and mechanical sensitivity in soft matter, much more than what would be possible with a single variable. One can easily imagine, however, that also other combinations of variables can be used to obtain mechanical control at a similar level.

Interestingly, for sufficiently stressed gels, we see that for the concentration, length and temperature series, these dependencies disappear and nearly universal behaviour emerges. Considering that cells can effectively apply stresses up to 10 Pa to their local environment^3^,^13^, it is even more intriguing to see that for PIC gels and for many biological gels, this ‘universal’ transition occurs well into the biologically accessible range. This may have implications for how we currently consider the role of mechanics in, for instance, tissue engineering. Cells may be able to control their own micromechanical environment by adhering to the matrix and actively stretching it. The observed universal behaviour of variables such as, in particular, concentration but also polymer length suggests that these parameters are ineffective for mechanical control, but merely decrease the porosity of the matrix, potentially reducing efficient nutrient transport and inhibiting cell spreading and proliferation. This supports the idea that, in fact, cells may probe different material properties than those which one characterizes with straightforward linear mechanical analysis^11^,^12^.

In conclusion, we described the effect of common, easily controllable variables (concentration, polymer length and temperature) on the mechanical properties of semi-flexible polymer hydrogels. Such gels are highly strain stiffening and we can use this property to create extremely stress-sensitive materials. We anticipate that the behaviour that we described is ubiquitous, in other words, many other strain-stiffening materials will behave similarly. Why, then, were these properties never described before? Two major reasons: (1) the majority of strain-stiffening materials have biological origins and it is often not easy to precisely define and control critical parameters such as length or connectivity, and (2) the length scales in the materials should match to enter the highly responsive regime: both the length (*L*) and the stiffness of the polymer bundle or filament (*l*_p_) should be of the same order of magnitude as the mesh size of the network. When the polymers are too flexible (*l*_p_ is too low), linear behaviour dominates and stiffening will only be observed at high stresses where the gel is likely to break. In addition, a (significant) minimum length is required to maintain network properties (sufficient crosslinks) at low concentrations. Typically, the persistence length of synthetic polymers is too low, even when we consider them to be rigid polymers. We firmly believe that bundle formation is the best way forward to increase *l*_p_, but full control over such process remains a challenge to be solved. The outlook, however, is a class of materials where the mechanical properties (*G*_0_, *σ*_c_ and *m*) can be readily adjusted by changing the conditions (*c*, *L*, *T*, and more), but also can be dramatically and immediately altered as a function of stress and/or other external stimuli. Such tremendous level of control will be highly beneficial for the biomedical field as well as other fields that use responsive soft matter applications.

## Methods

### Polymer synthesis and characterization

The isocyanide monomer^18^ was dissolved in freshly distilled toluene (50 mg ml^−1^) and stirred for several minutes. A solution of Ni(ClO_4_)_2_.6H_2_O (0.1 mg ml^−1^) in freshly distilled toluene/ethanol (9:1) was added at once. The mixture was stirred for 24 h at room temperature under air. The resulting polymer was precipitated in diisopropyl ether with vigorous stirring and collected by filtration. The solid was redissolved in dichloromethane and again precipitated in diisopropyl ether and collected by filtration. This procedure was repeated one more time and the polymer was dried under vacuum to yield the polymer as a yellow solid in 60–70% yield. The molecular weight of the polymer was characterized by viscometry and AFM, as shown in Supplementary Information. For viscometry measurements, the polymer was dissolved in acetonitrile (0.02–6.0 mg ml^−1^) and the intrinsic viscosity of these solutions was measured at a temperature of 25 °C. The average viscosity molecular weight, *M*_v_, of the polymers was calculated using the empirical Mark–Houwink equation, 
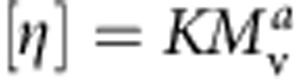
, where [*η*] is the intrinsic viscosity of the polymer solution as determined from viscometry measurements (Supplementary Fig. 3), and Mark–Houwink parameters *K* and *a* depend on polymer and solvent characteristics. We used values that were previously determined for (other) rigid polyisocyanides^26^: *K*=1.4 × 10^−9^ and *a*=1.75. For AFM measurements, the polymer was dissolved in freshly distilled dichloromethane (10^−3^ mg ml^−1^). This polymer solution was spin coated onto a freshly cleaved Muscovite Mica surface at 3,000 r.p.m. AFM measurements were performed using a dimension 3100 microscope operated with a nanoscope IV control unit (Digital Instruments). All images were recorded with the AFM operating in Tapping Mode in air at room temperature, with a resolution of 1,024 × 1,024 pixels. Commercial tapping-mode golden-coated silicon tips (NT-MDT) were used with a typical resonance frequency around 300 kHz. Polymer lengths were evaluated using ImageJ software.

### Rheology

Rheological measurements were carried out with a stress-controlled rheometer (Discovery HR-1, TA Instruments) in an aluminium parallel plate geometry (40 mm diameter) with a gap of 500 μm in a temperature-controlled environment. To probe the linear regime (*G*_0_), the sample was heated to the desired temperature, and after a short waiting period for equilibration the complex modulus G* was determined by applying an oscillating deformation of amplitude *γ*=0.01 in a frequency sweep of *ω*=10–0.1 Hz. The nonlinear regime (*σ*_c_ and *m*) was studied using a pre-stress protocol, where the sample, at the desired temperature was subjected to a constant stress *σ*_0_ with a small oscillatory stress superposed, also at *ω*=10–0.1 Hz. In the pre-stress protocol that we use to probe the nonlinear mechanical regime, we apply a constant stress to the material. We did not find relaxation processes in the materials, even at high stresses.

## Author contributions

M.J. and M.F.J.M. synthesized the materials; M.D. performed simulation studies. M.J. and P.H.J.K. designed, conducted and interpreted the rheological experiments. F.C.M., A.E.R. and P.H.J.K. supervised the project. All authors contributed to the manuscript.

## Additional information

**How to cite this article:** Jaspers, M. *et al.* Ultra-responsive soft matter from strain-stiffening hydrogels. *Nat. Commun.* 5:5808 doi: 10.1038/ncomms6808 (2014).

## Supplementary Material

Supplementary Information Supplementary Figures 1-3

## Figures and Tables

**Table 1:** Molecular weights M_v_, distributions and lengths of the investigated PIC polymers.

	*M*_v_ (kg mol^−1^)	PDI*	*n*†	*L*‡ (nm)
**P1a**	86	—	272	34
**P1b**	140	—	443	55
**P1c**	195	1.3	617	77
**P1d**	277	1.4	877	110
**P1e**	339	1.4	1073	134
**P1f**	400	1.4	1266	158
**P1g**	452	1.3	1430	179

AFM, atomic force microscopy; PDI, polymer dispersity index

^*^The PDI, showing that the molecular weight distribution of the polymers was similar for all materials and was determined by statistical analysis of the AFM images. The PDIs of the shorter polymers could not reliably be obtained using this method.

^†^Degree of polymerization.

^‡^The calculated polymer contour length is based on the dimensions of the helical structure of the polymer^19^. As an example, the viscosity molecular weight *M*_v_=400 kg mol^−1^ of **P1f** corresponds to chains of *n*≈1,300 monomers with a contour length *L*≈160 nm.
