# Supplementary material for: Ultra-responsive soft matter from strain-stiffening hydrogels
Source: Nat Commun. 2014 Dec 16;5:5808. doi: 10.1038/ncomms6808 (PMC4275588; doi:10.1038/ncomms6808)
Supplement: Supplementary Information — Supplementary Figures 1-3 [file ncomms6808-s1.pdf]

SUPPLEMENTARY MATERIAL

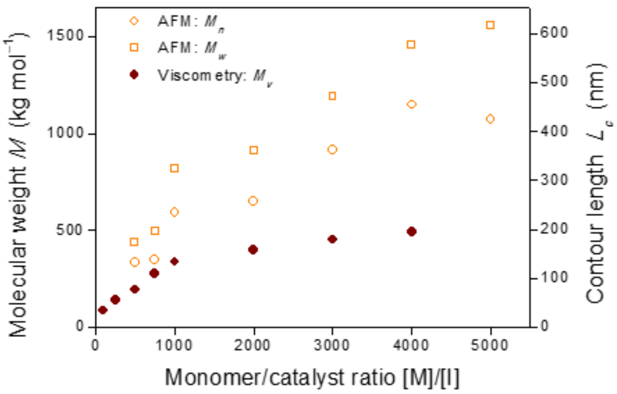

Supplementary Figure 1 | Molecular weight of the polymers as a function of the monomer/catalyst ratio used during the polymerization reaction. Solid dots correspond to M_v, determined by viscometry, open dots and squares correspond to M_n and M_w respectively, determined by AFM imaging.

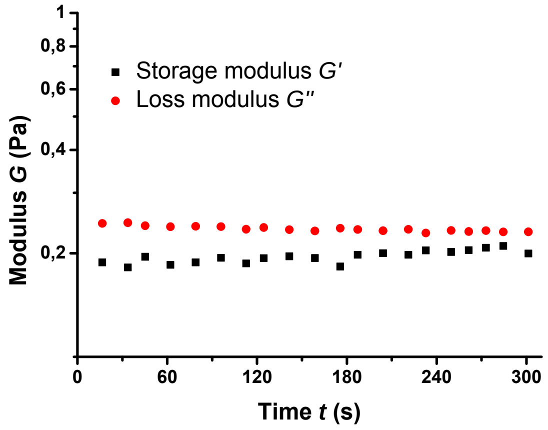

Supplementary Figure 2 | Linear regime of a 1f hydrogel at T = 17 °C. Recorded at a frequency of 1 Hz and a strain of 4%.

P1a:

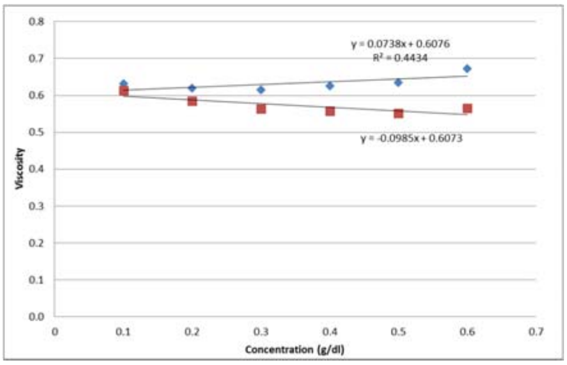

P1b:

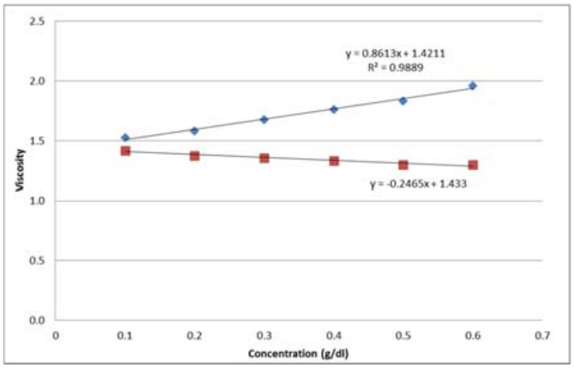

P1c:

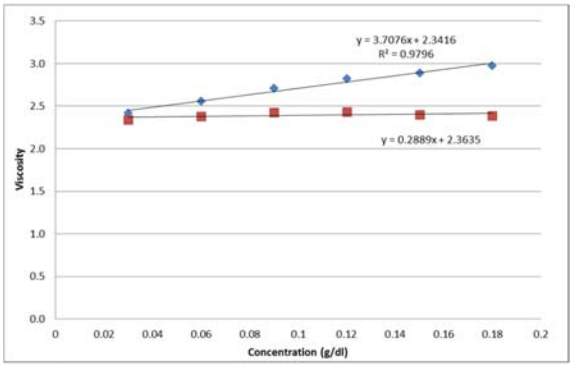

P1d:

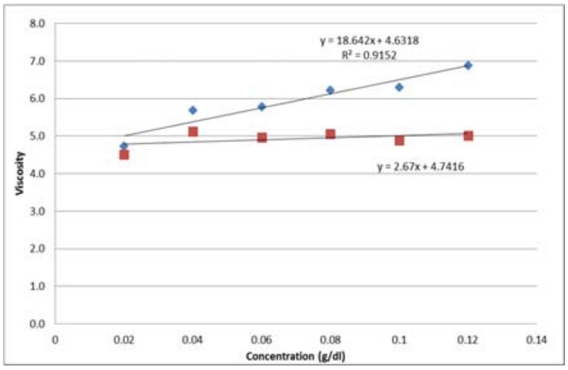

P1e:

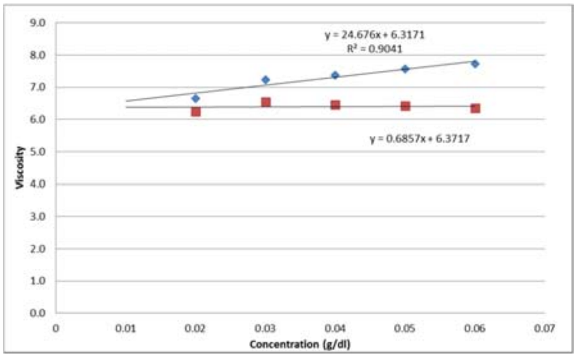

P1f:

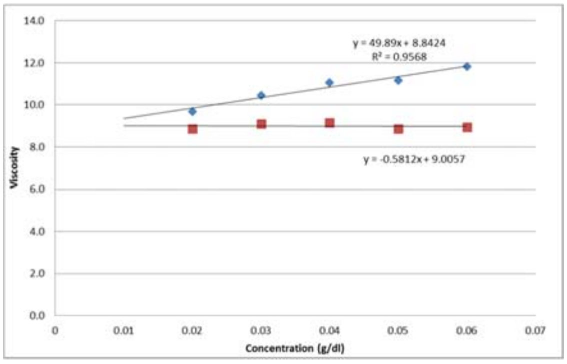

P1g:

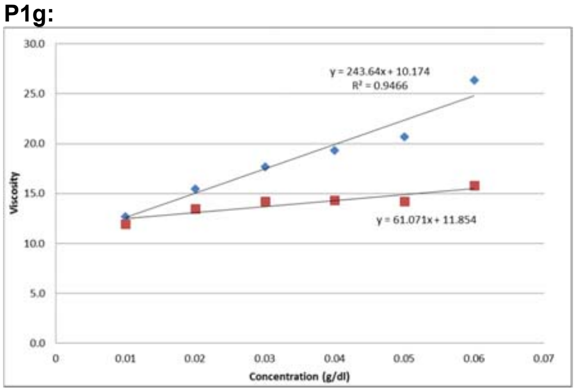

Supplementary Figure 3 | Viscometry data of P1a-P1g. Blue diamonds represent the reduced viscosity and red squares represent the inherent viscosity. The intrinsic viscosity was obtained from extrapolation to c = 0 g/dL. From the intrinsic viscosity, M_v was calculated using the Mark-Houwink equation with K and a values of $1.4 \cdot 10^{-9}$ and 1.75, respectively.
